# Molecular epidemiology and clinical features of hand, foot and mouth disease requiring hospitalization after the use of enterovirus A71 inactivated vaccine in chengdu, China, 2017-2022: a descriptive study

**DOI:** 10.1080/22221751.2022.2125346

**Published:** 2022-10-26

**Authors:** Xiaoxia Duan, Chaoyong Zhang, Xiao Wang, Xueling Ren, Hongxia Peng, Xueqin Tang, Liangzhi Zhang, Zhenhua Chen, Yan Ye, Mengmou Zheng, Wanzhen Zhong, Xiyue Chen, Yilan Zeng, Ping Yuan, Lu Long

**Affiliations:** a Department of Epidemiology and Health Statistics, West China School of Public Health and West China Fourth Hospital, Sichuan University, Sichuan, People’s Republic of China; b Public Health Clinical Center of Chengdu, Sichuan, People’s Republic of China; c Department of Immunization Program, Chengdu Municipal Center for Disease Control and Prevention, Sichuan, People’s Republic of China; d Department of Microbiology Laboratory, Chengdu Municipal Center for Disease Control and Prevention, Sichuan, People’s Republic of China

**Keywords:** Hand foot and mouth disease, molecular epidemiology, enterovirus, EV-A71 vaccine, clinical features

## Abstract

Three inactivated enterovirus A71 (EV-A71) vaccines have been widely vaccinated among children in the targeted age group in mainland China since mid-2016. However, comprehensive virological surveillance of hand, foot and mouth disease (HFMD) over multiple years after the use of EV-A71 vaccines has rarely been conducted. Using long-term data extracted from the Public Health and Clinical Center of Chengdu, we described the clinical, aetiological, and epidemiological characteristics of HFMD inpatients after the use of EV-A71 vaccines from 2017 through 2022. A total of 5115 patients were selected for analysis with a male-to-female ratio of 1.63:1 and were mostly under 5 years of age (97.6%). Among these cases, 4.3% presented with severe symptoms, and 4.1% of severe cases experienced significant complications. EV-A71 was no longer the major serotype for laboratory-confirmed HFMD, responsible for 15.6% of severe cases and 1.2% of mild cases. A significant downwards trend of EV-A71 infections was observed after the use of EV-A71 vaccines (P for trend < 0.001). Coxsackievirus A6 was the predominant pathogen, accounting for 63.5% of mild cases and 36.2% of severe cases. Coxsackievirus A10 (CV-A10) and A16 were sporadically detected, and an upwards trend was observed in the proportion of CV-A10 infections. This study provides baseline molecular epidemiology for the evaluation of EV-A71 vaccination impact and potential serotype replacement based on HFMD inpatients. Additional nationwide and population-based epidemiologic and serologic studies are essential to elucidate HFMD dynamics after the use of EV-A71 vaccines, and to inform public health authorities to introduce optimized intervention strategies.

## Introduction

Hand, foot and mouth disease (HFMD) is a highly contagious childhood illness usually caused by a group of enteroviruses that comprise 15 species [1-3]. Enterovirus A71 (EV-A71) and coxsackievirus A16 (CV-A16) have historically been regarded as the most common causative pathogens for HFMD [4,5]. However, coxsackievirus A6 (CV-A6)- and coxsackievirus A10 (CV-A10)-associated HFMD cases have increased in most regions [6-8]. As such, these four serotypes have now become major pathogenic causes of HFMD and have been the most predominantly detected [9].

HFMD is mostly a self-limiting disease, while a small proportion can rapidly develop neurological and systemic complications [2]. EV-A71 is the dominant pathogen causing severe and critical cases [10,11]. Three licenced inactivated EV-A71 vaccines have been developed in China and have been widely inoculated among children in the targeted age group since mid-2016 [12,13]. After that, severe cases markedly decreased [14]; however, the incidence of HFMD did not decline, and non-EV-A71-related HFMD cases increased over time. More than 1.35 million HFMD cases with an incidence rate of 96.08 per 100,000 were reported in mainland China in 2021 and ranked first in the number of all notifiable infectious diseases [15]. As of June 2022, the cumulative total number of reported cases reached approximately 24.92 million since HFMD was listed as a notifiable infectious disease in 2008, 3701 of whom died according to the National Health Commission of the People’s Republic of China (http://www.nhc.gov.cn/).

HEMD inpatients brought a greater socioeconomic burden to families and society compared with that of mild outpatients. The summary of studies on the economic burden of HFMD in China illustrated that the total costs of mild inpatients were approximately 5 times higher than those of outpatients [16]. Additionally, current epidemiological investigations of HFMD inpatients were mostly conducted before the use of the EV-A71 vaccines in China [17,18]. A few short-term studies described the serotype distribution of HFMD inpatients after EV-A71 vaccination, but only a portion of patients were sampled for enterovirus nucleic acid testing [19,20]. Long-term epidemiological data on HFMD-associated EV serotypes after the use of the EV-A71 vaccine remain scarce in China. Comprehensive virological surveillance of inpatients with HFMD and monitoring of serotype shift patterns over multiple years have rarely been conducted. Given the higher substantial burden and complex epidemic dynamics of inpatients with HFMD, molecular epidemiology and clinical feature studies are essential to inform the development of multivalent vaccines and the introduction of appropriate interventions. Herein, we describe the clinical, aetiological, and epidemiological characteristics of HFMD based on long-term data collected from the only designated referral hospital for the diagnosis and treatment of HFMD in Chengdu from 2017 to 2022.

## Materials and methods

### Definitions

A probable case was defined as a case who presented with mouth vesicular exanthema/ulcers and rashes over the hands, feet, or buttocks with or without fever. A laboratory confirmed case was defined as a patient who was positive for EV detected by real-time reverse-transcription polymerase chain reaction (rRT‒PCR). A case was classified as severe if he or she presented with any symptoms related to neurological involvement, cardiopulmonary failure, or both. Otherwise, the patient was categorized as a mild case. The details of relevant definitions can be found in the guidelines [21].

### Study setting

This retrospective study aimed to describe the molecular epidemiological and clinical characteristics of inpatients with HFMD after the use of the EV-A71 vaccines in Chengdu city. This study was performed at the Public Health Clinical Center of Chengdu, which was the only non-profit hospital designated by the Chinese government for infectious diseases in Chengdu and throughout Sichuan Province. This hospital accepted the majority of HFMD inpatients in Chengdu every year. The criteria for admitting patients with HFMD to the hospital are consistent with warning indicators for deterioration and impending critical disease as indicated in the Chinese guidelines for the diagnosis and treatment of HFMD (2018 edition) [21]. In addition, HFMD patients diagnosed with neurological or cardiopulmonary complications were also admitted to the hospital.

### Study population

**Figure 1.**
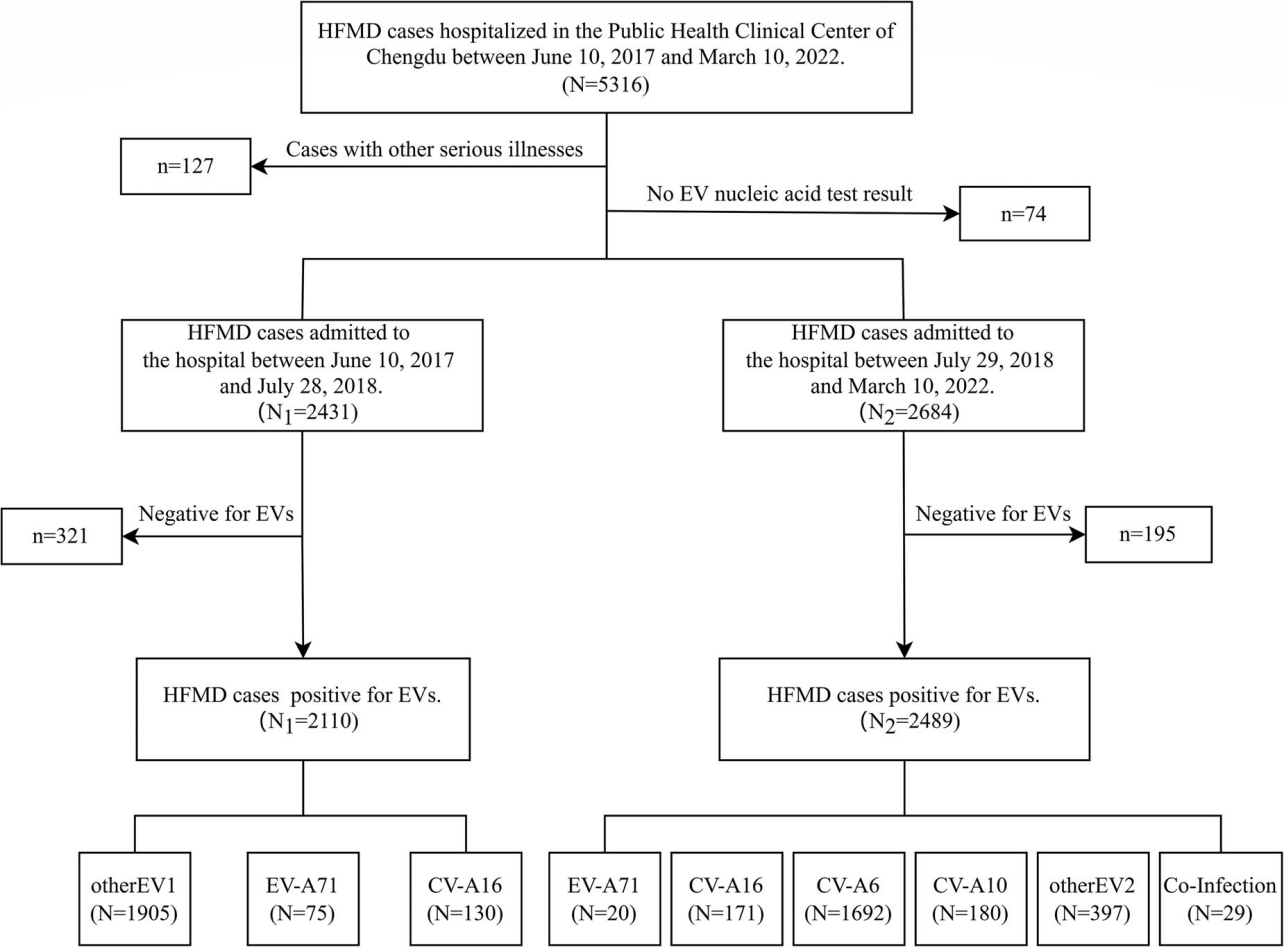
Flowchart illustrating the enrolled inpatients during the study period (from 10 June 2017–10 March 2022).

A total of 5316 patients with HFMD were admitted to the Public Health Clinical Center of Chengdu from 10 June 2017 to 10 March 2022. We excluded any patient with a primary discharge diagnosis of other serious disease. We also excluded any patient without enterovirus nucleic acid test results. A total of 5115 HFMD cases were finally selected for analysis, of whom 4599 were positive for enterovirus. The main components of the patient enrolment process are shown in Figure 1.

### Data collection

Throat swabs were collected from all admitted patients on the day of admission, and rRT‒PCR was performed within 24 h. In addition to general enterovirus, all patients were tested for EV-A71 and CV-A16 serotypes using a commercial rRT‒PCR Kit (BioPerfectus Technologies, Jiangsu, China) to confirm diagnosis, and CV-A6 and CV-A10 serotypes have been added since 29 July 2018. We extracted data from the clinical electronic medical records system via Epidata3.1 software using a data abstraction form developed based on previous studies. Clinical information, including enterovirus serotype, baseline characteristics, length of hospital stay, disease severity and outcome, was extracted by trained study staff from the electronic medical records.

### Statistical analysis

Quantitative variables were tested for normal (Gaussian) distribution using the Shapiro‒Wilk normality test, and all variables displayed a nonnormal distribution in this study. Nonnormally distributed continuous variables were expressed as the median (25th percentile (P_25_), 75th percentile (P_75_)) and compared across groups by the Mann‒Whitney U test or Kruskal‒Wallis rank sum test. Multiple comparison tests after the Kruskal‒Wallis test were conducted using the “kruskalmc” function in the “pgirmess” package [22]. Categorical variables were expressed as counts (n) and percentages (%) and compared across groups by the Chi-square test or Fisher’s exact probability test, as appropriate. The Cochran–Armitage test was implemented for trend analysis for the proportion of patients with different serotypes and patients with severe disease throughout the study period. All data were collected using Epidata 3.1 software and processed using Microsoft Excel (2021 MSO). Statistical analysis of the data was conducted with RStudio software (R version 4.1.2; https://www.r-project.org). All statistical tests reported two-sided *P* values; *P* < 0.05 was considered significant.

### Ethics statement

This study was approved by the Ethics Committee of the Public Health Clinical Center of Chengdu, and due to the nature of the retrospective study, the need for informed consent from individual patients was waived. No confidential information was involved in this research.

## Results

### Baseline characteristics of the patients

From 10 June 2017 through 10 March 2022, 5316 patients diagnosed with HFMD were admitted to the hospital, of whom 5115 patients with enterovirus nucleic acid test results and without other serious illness were selected for this study **(**Figure 1**)**. The median (P_25_, P_75_) age at admission was 19.28 (13.93, 28.32) months. Most patients (97.6% [4993]) were under 5 years of age, and 103 (2.0%) patients were under 6 months of age. Only seven adults (>18 years old) with HFMD were admitted to the hospital during the study period. Most patients were male, with a male-to-female ratio of 1.63 **(**Table 1**)**.

**Table 1.** Basic characteristics of 5115 inpatients with hand, foot, and mouth disease.

Characteristic	All (N=5115)	Mild (N=4897)	Severe (N=218)	P-value
Sex, n (%)				0.019**^a^**
Female	1947 (38.1)	1847 (37.7)	100 (45.9)	
Male	3168 (61.9)	3050 (62.3)	118 (54.1)	
Age, month, M (P25, P75)	19.28 (13.93, 28.32)	19.19 (13.80, 28.03)	22.51 (16.07, 36.72)	<0.001**^b^**
Age groups, month, n (%)				<0.001**^a^**
< 12	844 (16.5)	833 (17.0)	11 (5.0)	
12-23	2545 (49.8)	2436 (49.7)	109 (50.0)	
24-35	930 (18.2)	891 (18.2)	39 (17.9)	
≥ 36	796 (15.6)	737 (15.1)	59 (27.1)	
Hospitalization, day, M (P25, P75)	5.92 (4.98, 6.76)	5.88 (4.93, 6.71)	7.16 (6.56, 7.86)	<0.001**^b^**
LOS, day, n (%)				<0.001**^a^**
< 6	2683 (52.5)	2653 (54.2)	30 (13.8)	
≥ 6	2432 (47.5)	2244 (45.8)	188 (86.2)	
Patient source, n (%)				<0.001**^a^**
Emergency patients	1790 (35.0)	1750 (35.7)	40 (18.3)	
Outpatients	3325 (65.0)	3147 (64.3)	178 (81.7)	
Admission condition, n (%)				<0.001**^c^**
Urgent	210 (4.1)	183 (3.7)	27 (12.4)	
Critical	22 (0.4)	13 (0.3)	9 (4.1)	
General	4883 (95.5)	4701 (96.0)	182 (83.5)	
Outcome, n (%)				0.012**^a^**
Fully recovery	4438 (86.8)	4236 (86.5)	202 (92.7)	
Getting better	677 (13.2)	661 (13.5)	16 (7.3%)	

Notes: M, median; LOS, length of hospital stay; **^a^**Chi-square test; **^b^**Mann–Whitney U test; **^c^**Fisher’s exact probability test.

### Disease severity and clinical characteristics of the patients

A total of 4897 (95.7%) patients presented with mild symptoms, and 218 (4.3%) patients were classified as having severe disease during the study period. All patients were discharged smoothly, with no deaths. The percentages of cases with severe disease in total inpatients dropped from 49.1% (2017), 31.7%% (2018), 6.4% (2019) to 2.8% (2020) and 10.1% (2021), which was significantly decreased after the use of EV-A71 vaccines (P for trend < 0.001). There was a significant association with sex among different severities (*P* = 0.019). Children with mild HFMD were younger than those with severe HFMD (*P* < 0.001). Severe cases had longer hospital stays than mild cases (*P* < 0.001). The proportion of patients with urgent/critical admission conditions among severe cases (16.5%) was approximately 4.1 times greater than that among mild cases (4.0%) (*P* < 0.001). There was also a considerable difference in outcomes between mild and severe HFMD cases (*P* = 0.012) **(**Table 1**)**. Of the 218 severe cases, 204 (93.6%) cases received hormone treatment, 26 (11.9%) cases received intravenous immunoglobulin (IVIG) therapy, 1 (0.5%) case received orotracheal intubation, and none received mechanical ventilation or tracheostomy. Of the 218 severe cases, 9 (4.1%) cases had significant complications, including 6 (2.8%) with encephalitis, 1 (0.5%) with encephalomyelitis, 1 (0.5%) with pneumonia plus circulatory collapse, and 1 (0.5%) with pneumonia; however, they were not correlated with the EV serotypes (*P* = 0.748).

### Temporal patterns of enterovirus serotypes

Among 5115 patients with enterovirus nucleic acid test results, 4599 (89.9%) were laboratory-confirmed patients, while 516 (10.1%) clinically diagnosed patients were negative for enterovirus. The number of hospitalized patients with HFMD decreased yearly except in 2018 **(**Figure 2a**)**. The main prevalence peak of HFMD inpatients was April to July and persisted until August except in 2020 **(**Figure 2b**)**. One small peak was also observed from October to November **(**Figure 3a**)**. Between June 2017 and July 2018, 1905 of 2110 (90.3%) laboratory-confirmed HFMD cases were untyped, while the proportion of such cases dropped to approximately 20% since August 2018 **(**Figures 2b and 3b**)**.

**Figue 2.**
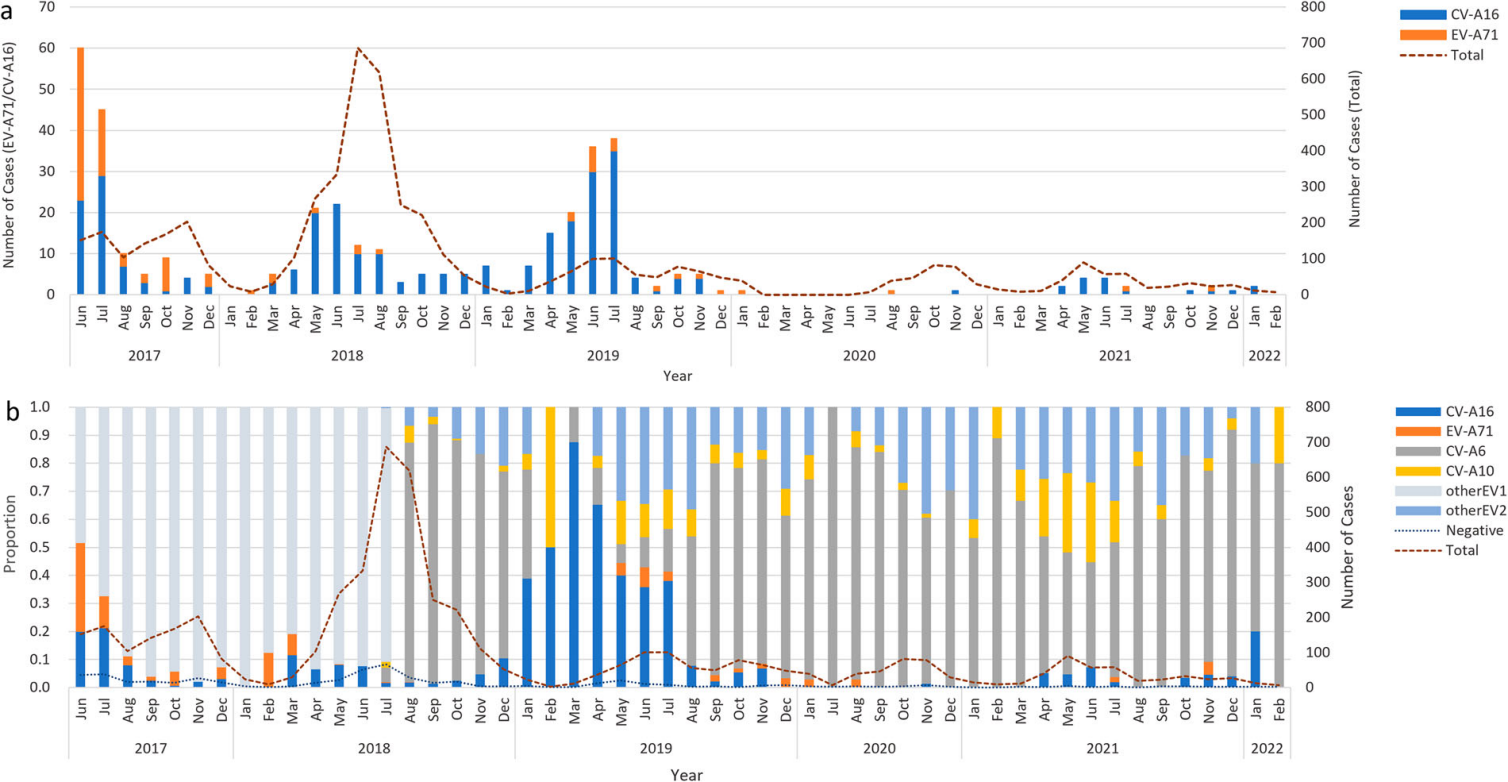
(a) The monthly distribution of all HFMD inpatients from 2017 to 2022 in the Public Health Clinical Center of Chengdu; (b) Serotype distribution of Enteroviruses positive HFMD inpatients in the Public Health Clinical Center of Chengdu.

**Figue 3.**
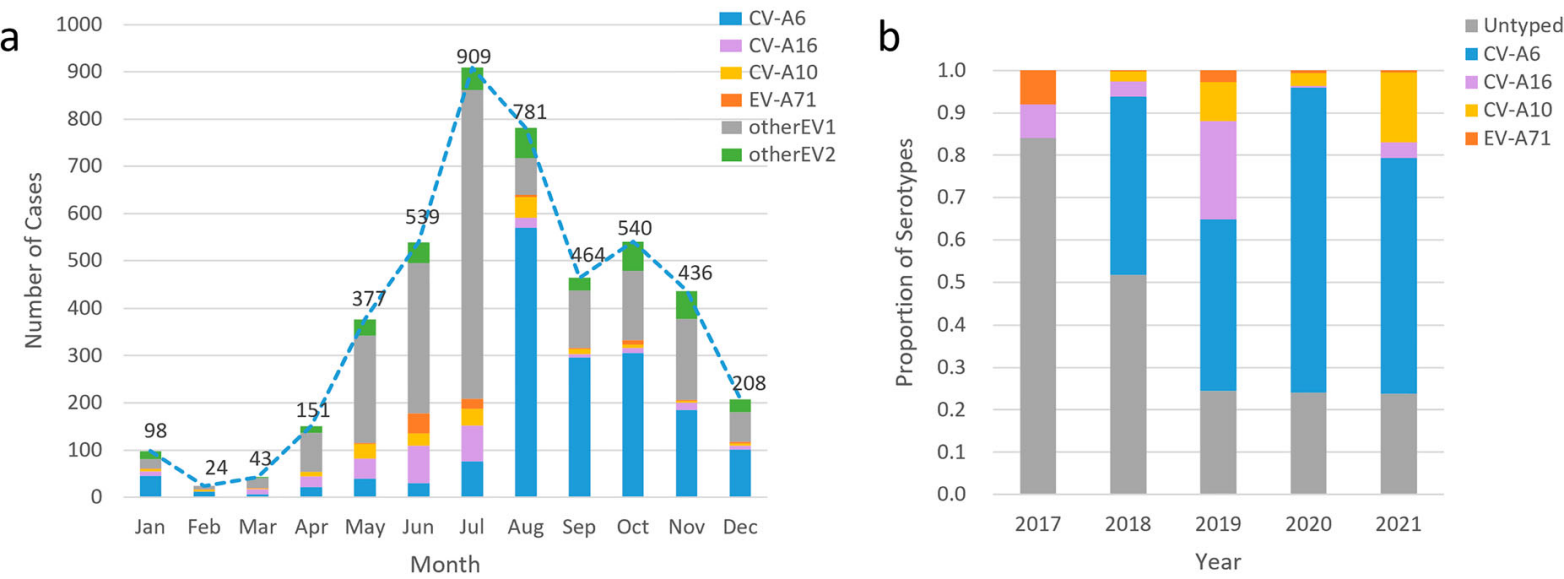
(a) The monthly serotype distribution of enteroviruses positive HFMD inpatients in the Public Health Clinical Center of Chengdu; (b) The yearly serotype distribution of all HFMD inpatients in the Public Health Clinical Center of Chengdu, 2017-2022.

Of 4599 laboratory-confirmed patients, 2268 (49.3%) cases were infected with one of the four predominant serotypes (including 1692 CV-A6 [74.6%], 301 CV-A16 [13.3%], 180 CV-A10 [7.9%], and only 95 EV-A71 [4.2%]). CV-A6 was the predominant serotype among laboratory-confirmed inpatients since it was detected, accounting for 81.7%, 39.1%, 71.6%, and 55.5% of such cases annually from the second half of 2018–2021 **(**Figure 3b**)**. The proportion of EV-A71-induced cases in total laboratory confirmed cases dropped sharply from 8.0% in 2017 to 0.3% in 2018 and then remained at a low prevalence level (2.7% in 2019, 0.7% in 2020 and 0.5% in 2021) **(**Supplementary Table S1). A significant downwards trend of EV-A71 was observed (P for trend < 0.001), although a small peak in admissions of EV-A71-related cases was observed in the summer of 2019 **(**Figure 4**)**. The number of CV-A16-infected cases remained at a similarly low level, accounting for approximately 10% of cases during the hospitalization peaks of each year except for the first half of 2019, in which 40% of inpatients were infected with CV-A16 **(**Figures 2b and 4**)**. The number of CV-A10-infected cases was consistently low, but an upwards trend was observed in the proportion of CV-A10-infected cases after the use of EV-A71 vaccines (P for trend < 0.001) **(**Supplementary Table S1). The monthly proportion distribution of different enterovirus serotypes from 2017 to 2021 is shown in supplementary Figure S1.

**Figue 4.**
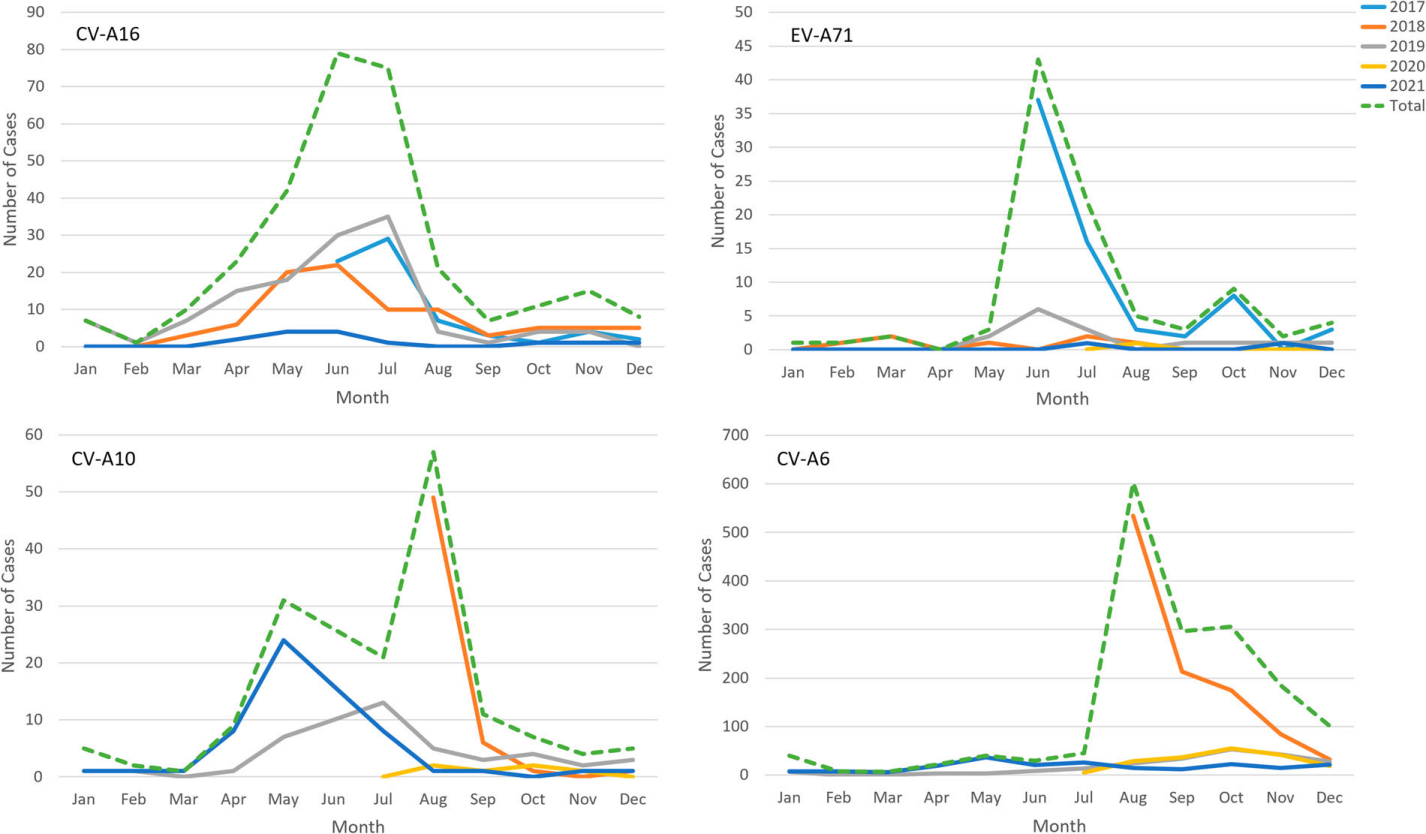
The monthly distribution of EV-A71-, CV-A16-, CV-A6- and CV-A10-associated HFMD inpatients from 2017 to 2021 in the Public Health Clinical Center of Chengdu.

### Serotype distribution among inpatients with mild and severe HFMD

There were considerable differences in the detection rates of EV-A71, CV-A6, CV-A10 and CV-A16 between patients with severe and mild HFMD. A total of 2268 (44.3%) inpatients were positive for one of the four predominant serotypes, among which EV-A71-infected patients were the most likely to develop severe disease (35.8%), followed by CV-A10 (4.4%), CV-A16 (3.7%), and CV-A6 (1.0%) **(**Figure 5a**)**. From 2017 to 2022, of the 4897 mild cases, 4035 (82.4%) patients were infected with nonEV-A71/CV-A16, 290 (5.9%) patients were positive for CV-A16, and only 61 (1.2%) patients were positive for EV-A71. Of 218 severe cases, 34 (15.6%) were infected with EV-A71, and 11 (5.0%) were infected with CV-A16 **(**Figure 5b**)**. Since 29 July 2018, among the 2637 mild cases, CV-A6 was the main pathogenic serotype, responsible for 63.5% of mild patients, followed by sporadic detection of CV-A10 (6.5%), CV-A16 (6.4%), and EV-A71 (0.7%). Of 47 severe cases, CV-A6 was also the predominant causative serotype (36.2%), followed by sporadic detection of CV-A10 (17.0%), CV-A16 (4.3%), and EV-A71 (2.1%) **(**Figure 5c**)**. The frequencies of the remaining serotypes detected in patients with mild/severe HFMD are shown in Figure 5.

**Figue 5.**
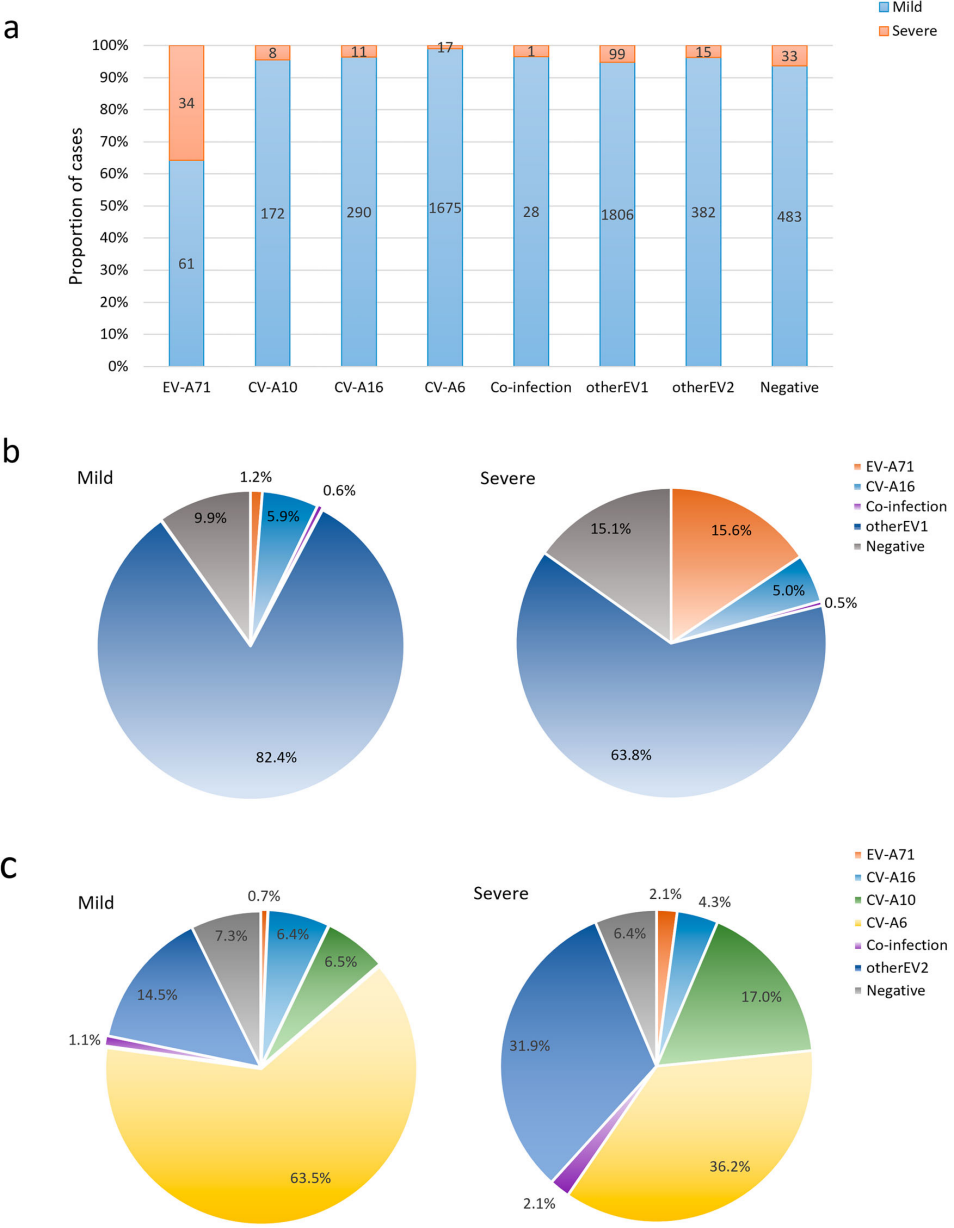
(a) Severity distribution of HFMD inpatients induced by different serotypes in the Public Health Clinical Center of Chengdu, 2017-2022; (b) Pie charts showing the detection rates of enterovirus serotypes in groups of patients with mild and severe HFMD from 10 June 2017 to 10 March 2022; (c) Pie charts showing the detection rates of enterovirus serotypes in groups of patients with mild and severe HFMD from 29 July 2018 to 10 March 2022.

### Serotype distribution among single EV-infected HFMD inpatients in different sex and age groups

A total of 4570 patients were infected by a single serotype, of whom 2845 were male patients and 1725 were female patients. Males outnumber females in all serotypes associated with HFMD cases, but there was no significant difference in sex composition between single-infected HFMD cases (*P* > 0.05). There was a significant difference in age composition between different serotype-infected HFMD cases (*P* < 0.001). CV-A6-associated HFMD patients were younger than patients induced by any of the other serotypes according to multiple comparisons, with a median (P_25_, P_75_) age of 18.37 (13.44, 24.99) months. EV-A71-associated HFMD patients were relatively older than patients infected with serotypes other than CV-A16, with a median (P_25_, P_75_) age of 33.78 (18.87, 51.50) months **(**Table 2**)**. The multiple comparison test results are shown in supplementary Table S2. The details of the age group distribution of cases with different serotypes and severities are shown in supplementary Figure S2.

**Table 2.** Serotype distribution among single EV-infected HFMD inpatients in different sex and age groups

Serotypes	Total (N=4570)	Sex			Age groups, months				
		Male (n=2845)	Female (n=1725)	MFR	M (IQR)	<12 (n=737)	12-23 (n=2323)	24-36 (n=832)	≥36 (n=678)
EV-A71	95	50 (1.8)	45 (2.6)	1.11	33.78 (18.87, 51.50)	9 (1.2)	28 (1.2)	13 (1.6)	45 (6.6)
CV-A16	301	188 (6.6)	113 (6.6)	1.66	26.67 (18.31, 40.34)	23 (3.1)	103 (4.4)	80 (9.6)	95 (14.0)
CV-A6	1692	1068 (37.5)	624 (36.2)	1.71	18.37 (13.44, 24.99)	308 (41.8)	907 (39.0)	286 (34.4)	191 (28.2)
CV-A10	180	104 (3.7)	76 (4.4)	1.37	20.27 (15.19, 28.89)	18 (2.4)	94 (4.0)	39 (4.7)	29 (4.3)
otherEV1	1905	1189 (41.8)	716 (41.5)	1.66	18.63 (13.58, 26.13)	327 (44.4)	1018 (43.8)	328 (39.4)	232 (34.2)
otherEV2	397	246 (8.6)	151 (8.8)	1.63	21.58 (15.54, 32.84)	52 (7.1)	173 (7.4)	86 (10.3)	86 (12.7)
P-value	0.320**^a^**				<0.001**^b^**	<0.001**^a^**			

Notes: ^a^Chi-square test; **^b^**Kruskal-Wallis rank sum test; MFR, male to female ratio; EV, enterovirus; CV, Coxsackievirus.

### Serotype distribution among coinfected HFMD inpatients in different sex and age groups

A total of 29 patients coinfected by 2 diverse serotypes of 4 predominantly detected enterovirus serotypes, including 12 female patients and 17 male patients, with a median (P_25_, P_75_) age of 22.48 (13.28, 33.09) months. There was no difference in sex composition (*P* > 0.05), or age composition (*P* > 0.05) between coinfected cases. The most frequent combination of serotypes was CV-A6 and CV-A10 (44.8%), followed by EV-A71 and CV-A10 (24.1%), EV-A71 and CV-A16 (10.3%), EV-A71 and CV-A6 (10.3%), CV-A16 and CV-A10 (6.9%), and CV-A16 and CV-A6 (3.4%). Of the 29 coinfected cases, 28 (96.6%) patients presented with mild symptoms, and only 1 (0.4%) female patient aged 16 months coinfected by EV-A71 and CV-A10 developed severe disease. Seventeen (58.6%) patients were coinfected by CV-A6 and another enterovirus, and 13 (44.8%) patients were coinfected by EV-A71 and one coxsackievirus **(**Table 3**)**.

**Table 3.** Serotype distribution among coinfected HFMD inpatients in different sex and age groups

Serotypes	Total (N=29)	Sex		Age groups, months			
		Female (n=12)	Male (n=17)	<12 (n=7)	12-23 (n=8)	24-36 (n=7)	≥36 (n=7)
CV-A6 & CV-A10	13	4 (33.3)	9 (52.9)	3 (42.9)	5 (62.5)	4 (57.1)	1 (14.3)
EV-A71 & CV-A10	7	5 (41.7)	2 (11.8)	2 (28.6)	**1^a^** (12.5)	1 (14.3)	3 (42.9)
EV-A71 & CV-A16	3	1 (8.3)	2 (11.8)	0	0	1 (14.3)	2 (28.6)
EV-A71 & CV-A6	3	1 (8.3)	2 (11.8)	2 (28.6)	1 (12.5)	0	0
CV-A16 & CV-A10	2	1 (8.3)	1 (5.9)	0	1 (12.5)	1 (14.3)	0
CV-A16 & CV-A6	1	0	1 (5.9)	0	0	0	1 (14.3)
P-value	-	0.586**^b^**		0.356**^b^**			

Notes: ^a^Case with severe disease; **^b^**Fisher’s exact probability test; EV, enterovirus; CV, Coxsackievirus.

## Discussion

In this study, the enterovirus serotype changes and clinical features of HFMD patients requiring hospitalization after the use of the EV-A71 vaccines were well demonstrated by the descriptive analyses. The total number of inpatients decreased by years except for 2018, in which an anomalously high HFMD incidence was also observed in several cities without known reasons in China [20,23,24]. Seasonal circulation patterns among EV-predominant serotypes revealed that inpatients had semi-annual outbreaks, as with all HFMD cases reported in Chengdu [25]. Notably, none of the HFMD patients were admitted to the hospital between February and June 2020, and the total number of cases reported in Sichuan Province was unusually low over the same period (https://www.sccdc.cn/). The primary reasons might be the strict prevention and control policies for the COVID-19 epidemic; for example, all schools, including off-campus training institutions, had not conducted any form of offline training or gathering activities according to documents issued by the Sichuan Provincial Department of Education (http://edu.sc.gov.cn/). Thus, children had fewer opportunities to participate in more activities and come into contact with children with latent infection or infected persons. The changes in public behaviours during the COVID-19 epidemic also played an important role [26].

As all children included in our study were hospitalized patients, our study showed a higher proportion of severe cases than studies based on surveillance data [4,27]. However, a previous study conducted in a hospital between 2014 and 2017 showed a critically higher proportion and case fatality of severe cases than our study (12.48% vs. 4.26%, 0.78% vs. 0.00%, respectively), with the most likely reason being that the EV-A71 vaccine had not yet been widely applied [28]. In general, EV-A71 was mainly responsible for the severe cases before the use of the EV-A71 vaccines in China [29-32]. But in the last few years, non-EV-A71 strains, such as CV-A6 and CV-A10, played an important role in both mild and severe cases, which illustrated a gradually increasing tendency [20,33,34]. In our study, the proportion of EV-A71- associated severe HFMD decreased to 15.6% during the whole study period and 2.2% after 29 July 2018. We speculated that the main reason for the smaller proportion of severe inpatients induced by EV-A71 was the rare number of severe cases and EV-A71 infections. In addition, the major circulating strains of EV-A71 were genotype C4a in China [29,35]. The widespread use of the EV-A71 vaccines might affect the landscape of other genotypes in circulation, but the sub-genotype switch of EV-A71 has not been observed or reported in China [36,37]. Further studies on EV-A71 genome characteristics based on the national surveillance are still useful and essential for monitoring the emergence of new variants and preventing HFMD outbreaks.

This long-term investigation showed that EV-A71-induced inpatients decreased dramatically after the use of EV-A71 vaccines, and the application of the EV-A71 vaccine might have a significant effect in children in Chengdu. We have also demonstrated that CV-A6 was a major cause of HFMD both in mild and severe inpatients after the implementation of EV-A71 vaccination in Chengdu. In fact, CV-A6 led to many outbreaks of HFMD locally and abroad before EV-A71 vaccination [38-40], but it was not added to detection tests before 2018, which might be the major reason for the unresolved serotypes in our study. Nationwide and population-based epidemiologic and serologic surveillance for enterovirus strains remains essential to elucidate the spectrum switch of enterovirus serotypes after the use of EV-A71 vaccines, as well as to inform the introduction of optimized intervention strategies to reduce the burden of HFMD. The results also indicate that the research and development of multivalent vaccines is urgently needed to prevent and control emerging enterovirus infections.

This study had several limitations. First, our results may carry the risk of false negatives because of testing only upon throat swabs, whereas previous studies have demonstrated that PCR testing based on stool samples and rectal swabs of HFMD patients has yielded high detection rates [18,41]. Second, this analysis included only hospitalized patients and these findings may not be generalizable to outpatients. Third, in the absence of electronic medical records before 10 June 2017, changes in serotype distribution prior to this date are unknown. Fourth, the prevalence of HFMD requiring hospitalization might be underestimated in this study due to the implementation of strict prevention and control policies in China during the COVID-19 epidemic. Finally, routine testing for CVA-10 and CV-A6 serotypes was not conducted in the hospital before July 2018, and the lack of further detection for patients infected with pan-enteroviruses may lead to biased observations of serotype distribution.

## Geolocation information

This study was conducted in No.16, Section 3, Renmin south road, Wuhou District, Department of Epidemiology and Health Statistics, West China School of Public Health and West China Fourth Hospital, Sichuan University, Chengdu 610041, Sichuan, China.

## Supplementary Material

Supplemental Material
