# Supplementary material for: Molecular epidemiology and clinical features of hand, foot and mouth disease requiring hospitalization after the use of enterovirus A71 inactivated vaccine in chengdu, China, 2017-2022: a descriptive study
Source: Emerg Microbes Infect. 2022 Oct 26;11(1):2510–9. doi: 10.1080/22221751.2022.2125346 (PMC9621254; doi:10.1080/22221751.2022.2125346)
Supplement: Supplemental Material [file TEMI_A_2125346_SM5947.docx]

**Supplementary**

**Table S1.** Trends in the proportion of different serotypes detected among laboratory-confirmed patients. (The Cochran-Armitage Trend test results)

| Serotypes | 2017 (N=864) | 2018 (N=2482) | 2019 (N=560) | 2020 (N=296) | 2021 (N=382) | P for trend |
| --- | --- | --- | --- | --- | --- | --- |
| EV-A71 | 69 (7.99) | 7 (0.28) | 15 (2.68) | 2 (0.68) | 2 (0.52) | <0.001 |
| CV-A16 | 69 (7.99) | 89 (3.59) | 126 (22.50) | 1 (0.34) | 14 (3.66) | 0.750 |
| CV-A10 | — | 57 (4.48) * | 50 (8.93) | 9 (3.04) | 63 (16.49) | <0.001 |
| CV-A6 | — | 1039 (81.68) * | 219 (39.11) | 212 (71.62) | 212 (55.50) | <0.001 |

Note: * Total number of cases was counted from 29 July to 31 December 2018, N=1272.

**Table S2.** Multiple comparison test results after Kruskal-Wallis (Significant. Level=0.05)

|  | obs.dif | critical.dif | difference |
| --- | --- | --- | --- |
| CV-A10 *vs.* CVA-16 | 533.8632 | 364.8915 | TRUE |
| CV-A10 *vs.* CV-A6 | 317.7968 | 303.6178 | TRUE |
| CV-A10 *vs.* EV-A71 | 671.5956 | 491.1103 | TRUE |
| CV-A10 *vs.* otherEV1 | 274.576 | 301.9813 | FALSE |
| CV-A10 *vs.* otherEV2 | 107.1844 | 347.9903 | FALSE |
| CV-A16 *vs.* CV-A6 | 851.66 | 242.2596 | TRUE |
| CV-A16 *vs.* EV-A71 | 137.7324 | 455.7359 | FALSE |
| CV-A16 *vs.* otherEV1 | 808.4392 | 240.2054 | TRUE |
| CV-A16 *vs.* otherEV2 | 426.6788 | 295.9783 | TRUE |
| CV-A6 *vs.* EV-A71 | 989.3924 | 408.3297 | TRUE |
| CV-A6 *vs.* otherEV1 | 43.22082 | 129.3699 | FALSE |
| CV-A6 *vs.* otherEV2 | 424.9812 | 215.9656 | TRUE |
| EV-A71 *vs.* otherEV1 | 946.1716 | 407.1144 | TRUE |
| EV-A71 *vs.* otherEV2 | 564.4112 | 442.3196 | TRUE |
| otherEV1 *vs.* otherEV2 | 381.7604 | 213.6587 | TRUE |

**Notes:**

Obs.df: Comparing the difference between the mean rank sums of the two groups;

Critical.dif: Comparing critical values between two groups;

FALSE indicates that the difference between the average rank sums of the two groups is less than the critical value, and the difference is not statistically significant;

TRUE indicates that the difference between the average rank sums of the two groups is greater than the critical value, and the difference is statistically significant.


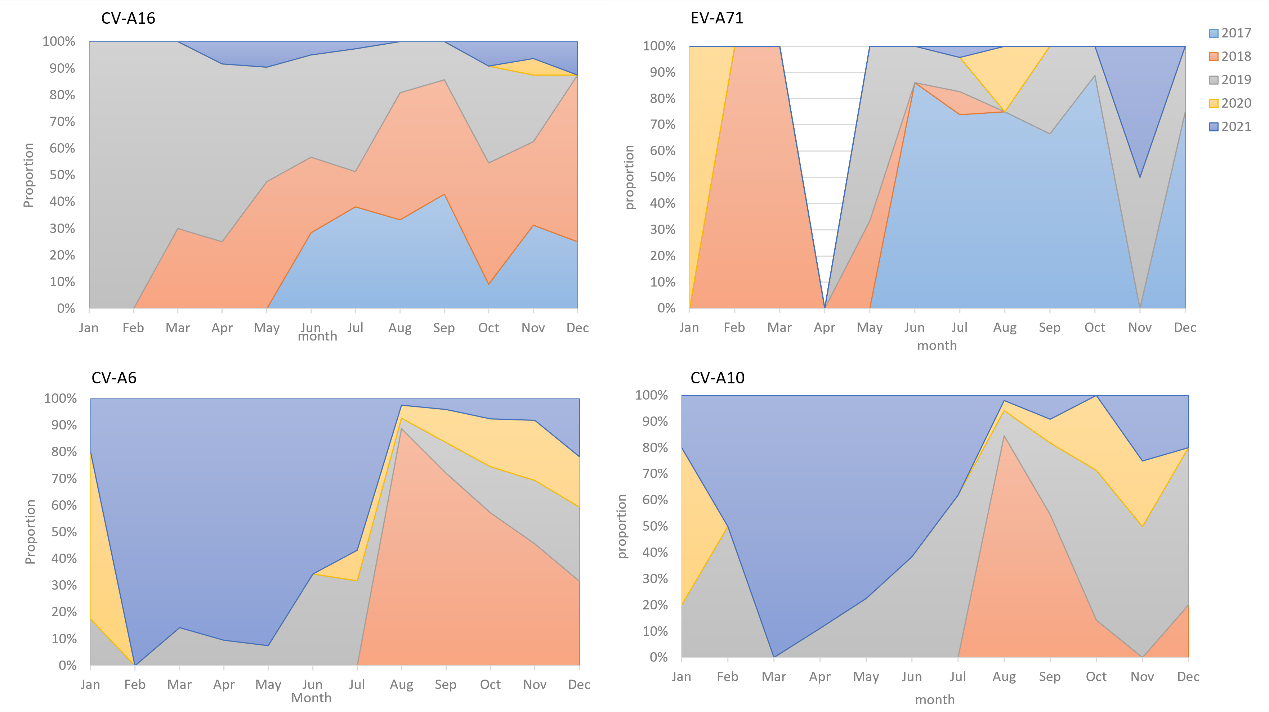


**Figure S1**. Monthly proportion distribution of cases induced by different enterovirus serotypes in Chengdu, 2017 - 2021.


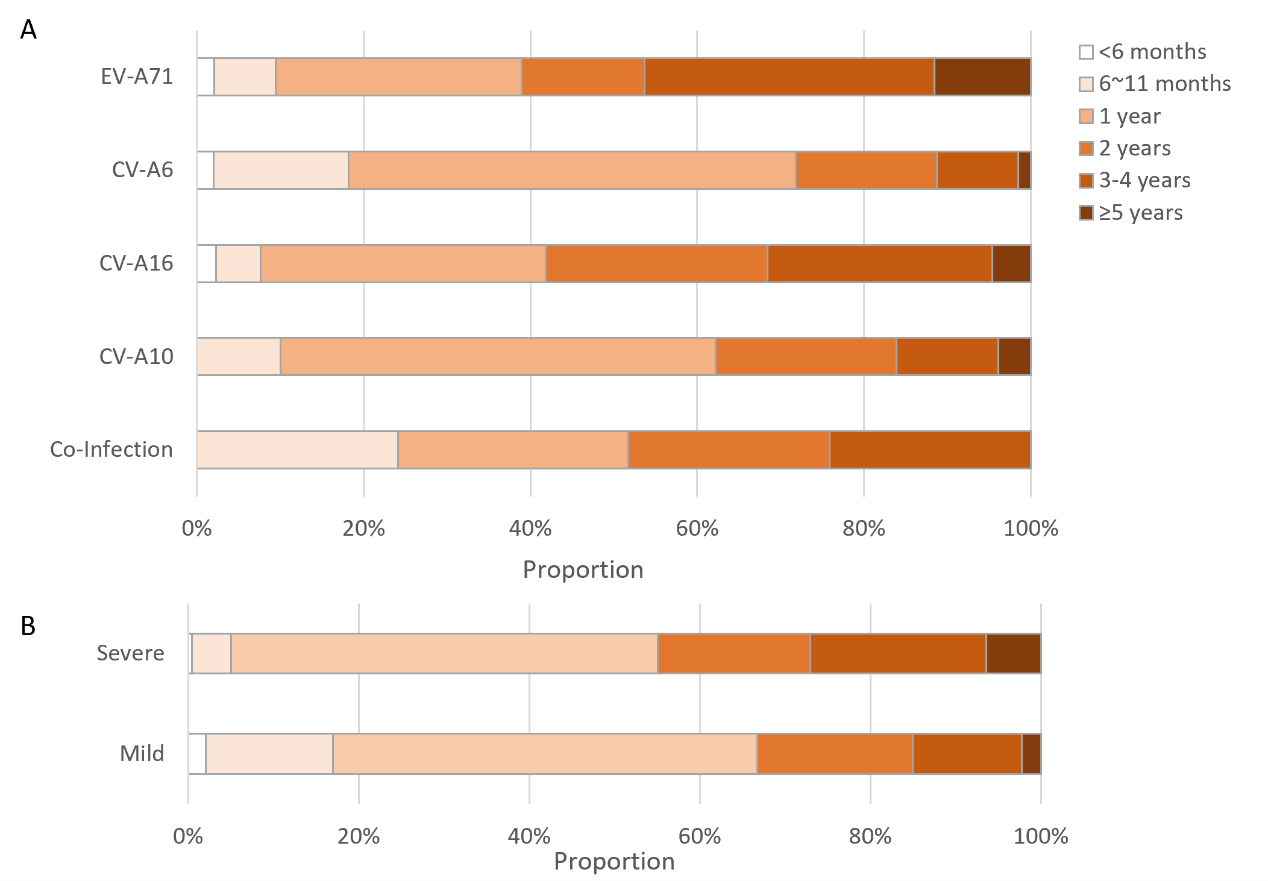


**Figure S2**. The details of age group distribution of cases induced by different serotypes and cases with different severity.
